# Modafinil Effects on Behavior and Oxidative Damage Parameters in Brain of Wistar Rats

**DOI:** 10.1155/2014/917246

**Published:** 2014-11-06

**Authors:** Felipe Ornell, Samira S. Valvassori, Amanda V. Steckert, Pedro F. Deroza, Wilson R. Resende, Roger B. Varela, João Quevedo

**Affiliations:** ^1^Laboratório de Neurociências, Programa de Pós-Graduação em Ciências da Saúde, Unidade Acadêmica de Ciências da Saúde, Universidade do Extremo Sul Catarinense, 88806000 Criciúma, SC, Brazil; ^2^Center for Experimental Models in Psychiatry, Department of Psychiatry and Behavioral Sciences, The University of Texas Medical School at Houston, Houston, TX 77054, USA

## Abstract

The effects of modafinil (MD) on behavioral and oxidative damage to protein and lipid in the brain of rats were evaluated. Wistar rats were given a single administration by gavage of water or MD (75, 150, or 300 mg/kg). Behavioral parameters were evaluated in open-field apparatus 1, 2, and 3 h after drug administration. Thiobarbituric acid reactive substances (TBARS) and protein carbonyl formation were measured in the brain. MD increased locomotor activity at the highest dose 1 and 3 h after administration. MD administration at the dose of 300 mg/kg increased visits to the center of open-field 1 h after administration; however, 3 h after administration, all administered doses of MD increased visits to the open-field center. MD 300 mg/kg increased lipid damage in the amygdala, hippocampus, and striatum. Besides, MD increased protein damage in the prefrontal cortex, amygdala, and hippocampus; however, this effect varies depending on the dose administered. In contrast, the administration of MD 75 and 300 mg/kg decreased the protein damage in the striatum. This study demonstrated that the MD administration induces behavioral changes, which was depending on the dose used. In addition, the effects of MD on oxidative damage parameters seemed to be in specific brain region and doses.

## 1. Introduction

Modafinil (MD) is a nonamphetaminergic psychoactive drug frequently prescribed for the treatment of sleep, such as narcolepsy, obstructive sleep apnea syndrome, and shift work sleep disorder [1]. Besides, it is well described in literature that MD enhances function in a number of cognitive domains as well as work memory and episodic memory [2–6]. These effects of MD on memory have also been described in psychiatric patients, suggesting that this drug is an excellent candidate agent for treatment of cognitive dysfunction in psychiatric disorders [7–9]. In addition, clinical research has shown that MD improves symptoms in patients with major depression, bipolar disorder, schizophrenia, and attention-deficit/hyperactivity disorder (ADHD) [10–12].

The mechanism of action of MD is poorly understood; however, it is known that this drug has an important effect on catecholamines, serotonin, glutamate, gamma amino-butyric acid, orexin, and histamine systems in the brain [1]. Besides, studies show that MD inhibits the dopamine transporter, increasing the dopaminergic neurotransmission in the vigilance circuits [13, 14]. Psychostimulants such as amphetamine, which also act on various neurotransmitter systems, have been shown to have a pronounced effect on behavior, including the generation of fear, anxiety, and hyperactivity [15–18]. However, MD is less related to side effects such as hyperactivity, anxiety, jitteriness, or rebound effects than the traditional stimulants [19].

Several studies suggested that psychostimulants administration can lead to oxidative stress in rat brain. The brain is particularly vulnerable to reactive oxygen species (ROS) production because it metabolizes 20% of total body oxygen and has a limited amount of antioxidant capacity [20]. Chronic administration of psychostimulants such as methylphenidate, m-amphetamine, and d-amphetamine in rats induced increased superoxide production, oxidative damage to protein, lipid, and DNA, and changes in enzymes antioxidants and the mitochondrial respiratory chain complexes [18, 21–27].

Therefore, the aim of present study was evaluating the effect of MD on behavior and oxidative stress parameters in the hippocampus, prefrontal cortex, amygdala, and striatum of rats.

## 2. Experimental Methods

### 2.1. Animals

The subjects were adult male Wistar rats (weighting 250–350 g) obtained from our breeding colony. Animals were housed as five in a cage with food and water available* ad libitum* and were maintained on a 12 h light/dark cycle (lights on at 7:00 a.m.) at a temperature of 22 ± 1°C. All experimental procedures were performed in accordance with the approval of the local Ethics Committee in the use of animals at the Universidade do Extremo Sul Catarinense. All experiments were performed at the same time during the day to avoid circadian variations.

### 2.2. Drugs and Pharmacological Procedures

The MD (Libbs Farmacêutica Ltda) was suspended in the vehicle-vehicle: 1% methylcellulose in water. The solutions were prepared immediately before use and were protected from the light during the experimental session. The suspended solution was under agitation during all the injection period. The control group received the vehicle.

### 2.3. Experimental Design

The total number of rats used in this experiment was 40 (*n* = 10 animals per group). Animals received a single dose of MD (75, 100, or 300 mg/kg body weight) in a volume of 1 mL/kg, administered by gavage. Control group received vehicle in a volume of 1 mL/kg. Locomotor activity was measured 1, 2, and 3 h after the injection, and the rats were killed by decapitation right after the open-field task.

### 2.4. Locomotor Activity

Locomotor activity was assessed using the open-field task as previously described [18, 28]. This task was performed in a 40 × 60 cm open-field surrounded by 50 cm high walls, made of brown plywood, with the floor being divided into 9 equal squares by black lines. The animals were gently placed on the left rear rectangle and left free to explore the arena for 5 min. In the open-field test, the following behavioral parameters were assessed.

Crossings (locomotor activity/horizontal activity): the total number of squares crossed by rats in whole test period was counted.

Rearings (exploratory activity/vertical activity): the total erect posture of rats in whole test period was counted.

Visits to the center of open-field: the total number of visits to the centre of open-field was counted. A center square of 30 × 30 cm was defined as the “center” area of the field.

### 2.5. Measurement of Oxidative Damage Markers

Rats were treated with MD or water as described above and were killed by decapitation 3 h after the last injection and their brains were removed and dissected for evaluation of oxidative damage levels in the prefrontal cortex, amygdala, hippocampus, and striatum. TBARS and protein carbonyl formations were measured as previously described [29, 30].

### 2.6. Thiobarbituric Acid Reactive Substances (TBARS)

The formation of TBARS during an acid-heating reaction was measured as an index of ROS production, which is widely adopted as a sensitive method for measurement of lipid peroxidation, as previously described [29]. Briefly, the samples were mixed with 1 mL of trichloroacetic acid 10% (TCA) and 1 mL of thiobarbituric acid 0.67% (TBA) and then heated in a boiling water bath for 15 min. TBARS were determined by the absorbance at 535 nm. Results are expressed as MDA (malondialdehyde) equivalents (nmol/mg protein).

### 2.7. Measurement of Protein Carbonyls

The oxidative damage to proteins was assessed by the determination of carbonyl groups based on the reaction with dinitrophenylhydrazine (DNPH) as previously described [30]. Briefly, proteins were precipitated by the addition of 20% trichloroacetic acid and redissolved in DNPH and the absorbance read at 370 nm.

### 2.8. Statistical Analysis

All analyses were performed with the statistical package for social sciences version 19.0 (SPSS Inc., Chicago, IL, USA). All data are presented as mean ± SEM. Differences between groups in behavioral analysis were verified using repeated measures analysis of variance to access the time response curve, followed by Tukey's post hoc tests. To test differences between groups in biochemical analysis, we used ANOVA, followed by Tukey post hoc tests. In all experiments, *P* values < 0.05 were considered to indicate statistical significance.

## 3. Results

### 3.1. Behavior Analysis

**Figure 1:**
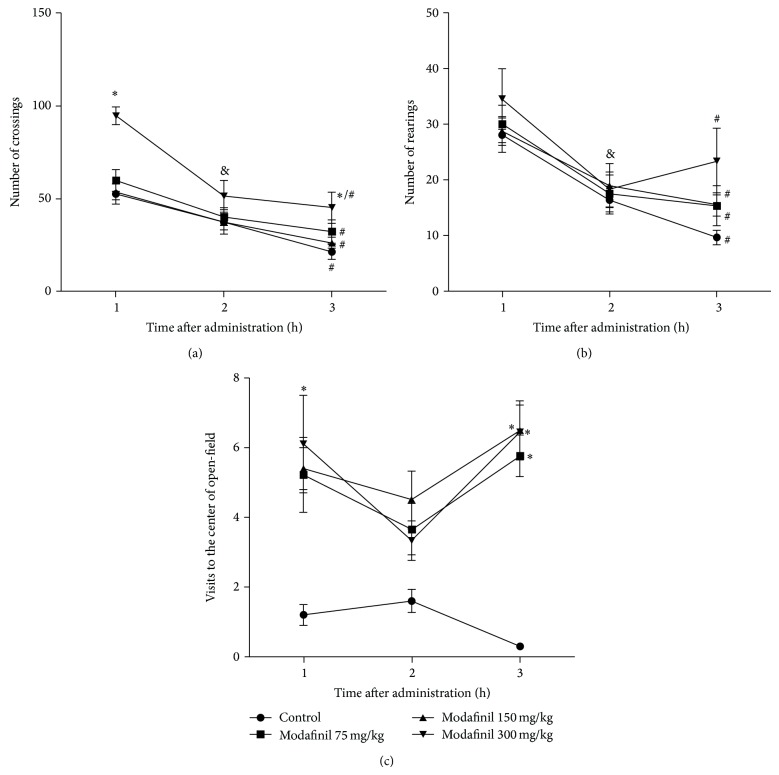
Effects of MD (75, 150, and 300 mg/kg) or water (control group) administration on number of crossings (a), rearings (b), and visits to the center of open-field (c) in rats subjected to the open-field test for 5 minutes. Behavior parameters were assessed in the open-field test three times: 1 hour and 2 and 3 hours after administration of MD or water. ^*^ *P* < 0.05 compared with control group. ^&^ *P* < 0.05 1 h versus 2 h. ^#^ *P* < 0.05 1 h versus 3 h. All analyses were performed according to repeated measures of variance, followed by the Tukey's test.

For the analysis of locomotion (crossings) (Figure 1(a)), the repeated measures analysis of variance revealed significant differences for MD administration (*F*(3.35) = 7.91, *P* < 0.001) and for the behavioral repetitions (*F*(2.7) = 54.82, *P* < 0.001). Further analysis with Tukey's post hoc test showed that MD at 300 mg/kg increased rat spontaneous locomotion compared to control group 1 h after administration. In addition, control group, MD at 75 mg/kg, and MD at 150 mg/kg displayed reduced number of crossings when reexposed 3 h later to the open-field, thus indicating habituation to the environment. However, MD at 300 mg/kg treated rats displayed reduced number of crossings when reexposed 2 and 3 h latter to the open-field. This difference may be explained by the motoric hyperactivity induced 1 h after MD administration at the dose of 300 mg/kg.

For the analysis of exploration (rearings) (Figure 1(b)), the repeated measures analysis of variance revealed significant differences for the behavioral repetitions (*F*(2.7) = 32.7, *P* < 0.001). Further analysis with Tukey's post hoc test showed that control group, MD at 75 mg/kg, and MD at 150 mg/kg displayed reduced number of rearings when reexposed 3 h latter to the open-field. MD at 300 mg/kg decreased the number of rearings when reexposed 2 and 3 h latter to the open-field.

For the analysis of visits to the center of open-field (Figure 1(c)), the repeated measures analysis of variance revealed significant differences for MD administration (*F*(3.34) = 15.70, *P* < 0.001). Further analysis with Tukey's post hoc test showed that MD at 300 mg/kg increased visits to the center of open-field compared to control 1 h after administration. In addition, MD at all doses administered increased visits to the center of open-field 3 h after administration.

### 3.2. Biochemical Analysis

**Figure 2:**
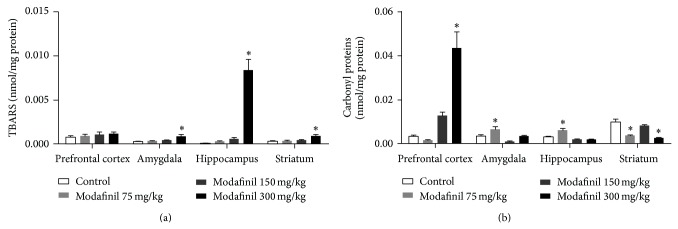
Effects of MD administration or water (control group) on TBARS (a) or protein carbonyl (b) levels in the prefrontal cortex, amygdala, hippocampus, and striatum of rats. ^*^ *P* < 0.05 versus control group, according to ANOVA followed by the Tukey's test.

As shown in Figure 2(a), TBARS levels were significantly increased in the amygdala (*F*(3) = 4.18, *P* = 0.014), hippocampus (*F*(3) = 44.9, *P* < 0.01), and striatum (*F*(3) = 7.07, *P* < 0.01) of rats treated with MD at 300 mg/kg as compared to control group.

As can be observed in Figure 2(b), a significant increase in carbonyl generation was detected after administration of MD in the prefrontal cortex (*F*(3) = 29.9, *P* < 0.01) at the dose of 300 mg/kg and in the amygdala (*F*(3) = 9.74, *P* < 0.01) and hippocampus (*F*(3) = 17.99, *P* < 0.01) at 75 mg/kg. Conversely, treatment with MD at 75 and 300 mg/kg significantly reduced carbonyl generation in the striatum (*F*(3) = 21.93, *P* < 0.01) as compared to control group.

## 4. Discussion

In the present study, we observed that a single injection of MD in a high dose (300 mg/kg) induces hyperlocomotion in rats, which does not remain 2 and 3 hours after administration. According to our results, MD significantly increased locomotor activity and increased striatal extracellular dopamine levels in rhesus monkeys [31]. Young and colleagues [14] showed that MD increased activity, rearing, and the smoothness of locomotor paths in C57BL/6J and 129/SJ mice. These behavior MD-induced alterations were related to increased synaptic dopamine and secondary actions mediated by dopamine drd1 and drd4 receptors. Unlike the above study, although there is a trend, we do not observe a significant increase in the exploratory behavior after MD administration. This discrepancy can be explained by differences in the methodology, species, and treatment time.

Here, we observed that control group and MD at low doses (75 and 150 mg/kg) reduced the number of crossings and rearings when reexposed 3 hours latter to the open-field, indicating habituation to the environment. MD at the high dose (300 mg/kg) reduced the number of crossings and rearings when reexposed 2 and 3 hours latter to the open-field. This discrepancy can be explained by the fact that 1 h after MD (300 mg/kg) administration increased significantly the number of crossings and a tendency to increase the number of rearings. Habituation to a novel environment is believed to be one of the most elementary forms of nonassociative learning. The repeated exposure to the same environment induces a reduction in the exploratory behavior, which can be taken as an index of habituation [32].

An interesting finding of the current study was that MD modulated anxiety-related behavior. In the open-field test, MD treated rats were less anxious and even tended to explore the aversive center area more than the controls. MD at 300 mg/kg increased the number of visits to the center of the open-field 1 h after administration. In addition, MD at all doses administered increased visits to the center of open-field 3 h after administration. In the literature, studies are controversial about the effect of MD on anxiety. Preclinical studies have shown either no effects of modafinil on anxiety [33, 34] or an anxiolytic effect [35]. Similarly, MD in the clinical studies shows either an anxiolytic effect [36] or no effect on anxiety [37, 38], while others demonstrate an anxiogenic effect [39, 40]. This difference between studies can be explained by variation in doses used (100 mg, 200 mg, or 400 mg) and in the dosing schedule (one time versus chronic dosing over a week or more). The anxiolytic-like effects of MD can be explained by its effects on the amygdala, which is a brain region implicated in anxiety, to threatening stimuli. A previous study showed that MD decreases amygdala reactivity to fearful stimuli [41]. It is known that the amygdala is rich in catecholaminergic and serotoninergic projections [42], and then probably MD reduces amygdala reactivity by changes in intra-amygdala signaling resulting from alterations in noradrenaline, dopamine, serotonin, or GABA systems [1, 43, 44] or from a combination of these effects.

In addition to inducing behavioral changes, it is well described in the literature that psychostimulants cause oxidative damage in both animal models [18, 26] and humans [45]. Our results show that MD increases oxidative damage to lipid and protein in the brain of rats. TBARS levels were increased in the amygdala, hippocampus, and striatum of rats treated with MD at high dose (300 mg/kg). In addition, we observed an increase in carbonyl generation after administration of MD in the prefrontal cortex at the dose of 300 mg/kg and in the amygdala and hippocampus at 75 mg/kg. Studies show that MD inhibits the dopamine transporter, increasing the dopaminergic neurotransmission [13, 14]. The increase in extracellular dopamine concentration induced by MD can induce overproduction of the toxic metabolite of dopamine oxidation [46, 47], leading to oxidative damage to proteins and lipids in the brain of rats. In the literature, there are no studies assessing brain injury after administration of MD; however, these studies are very important given that accessibility of MD, such as alertness-enhancing, memory-enhancing, and antifatigue drug, for healthy people is increasing [48].

Conversely, treatment with MD (75 and 300 mg/kg) reduced carbonyl generation in the striatum as compared to control group. Some studies have shown the striatal neuroprotective potential of MD. Previous studies show improvement survival of 1-methyl-1,2,3,6-tetrahydropyridine intoxicated dopaminergic neurons in the striatum after MD treatment, in animal model of Parkinson's disease [49–51]. Raineri and colleagues [52] have shown that MD administration attenuated methamphetamine-induced neurotoxicity in striatum of mice, suggesting a possible protective role of MD in this brain region. MD has been shown to improve learning in methamphetamine-dependent patients [53]. Results presented here suggest that MD administration might display antioxidant properties in the striatum; however, the protective effect of MD on the striatum is still unknown. Striatum integrates glutamatergic inputs from cortex and thalamus [54] with dopaminergic afferents from midbrain [55]. Dopamine signaling plays a preeminent role in striatal dependent learning [56] and in synaptic plasticity of the medium spiny projection neurons [57, 58]. Rossato and colleagues [59] showed that infusion of the D1 receptor agonist increased brain-derived neurotrophic factor (BDNF) levels and consequently synaptic plasticity. Thus, MD can be promoting synaptic plasticity by activating the dopaminergic system and consequently protecting the striatum against oxidative damage.

In conclusion, we are able to demonstrate that (1) MD induces hyperactivity at high dose (300 mg/kg) 1 h after administration, which does not remain 2 and 3 hours after administration. (2) MD showed anxiolytic effects in rats, increasing the number of visits to the center of the open-field. (3) MD induced oxidative damage to lipid and protein in the rat brain; however, the oxidative damage depends on the brain region analyzed and dose of MD administered. (4) Finally, MD could protect the striatum against protein oxidative damage. Caution must be taken when interpreting the results. First, antioxidant defenses were not measured; as this is the first study to examine the impact of MD on oxidative stress, we acknowledge that it could have helped in interpreting the results. However, second, MD was administered to healthy rats; the effects of MD on the oxidative damage in animal models of mental illness may show different results.
